# Concomitant motor responses facilitate the acquisition of multiple prior distributions in human coincidence timing

**DOI:** 10.1098/rspb.2024.2438

**Published:** 2025-01-29

**Authors:** Shu Natsume, Neil W. Roach, Makoto Miyazaki

**Affiliations:** ^1^ Graduate School of Integrated Science and Technology, Shizuoka University, Hamamatsu 432-8011, Japan; ^2^ School of Psychology, University of Nottingham, Nottingham NG7 2RD, UK; ^3^ Faculty of Informatics, Shizuoka University, Hamamatsu 432-8011, Japan

**Keywords:** timing, Bayesian estimation, multiple priors, concomitant motor response, vocalization

## Abstract

The brain optimizes timing behaviour by acquiring a prior distribution of target timing and integrating it with sensory inputs. Real events have distinct temporal statistics (e.g. fastball/slowball in ball sports), making it vital to acquire multiple prior distributions. In previous studies, participants acquired two prior distributions by assigning different types of motor responses or motor effectors to each prior. However, in daily tasks, different types of motor responses or effectors cannot always be selected for each target state. Here, we demonstrate that concomitant motor responses (CMRs) can facilitate multiple-prior acquisition. The non-CMR group made timing responses using only their dominant hand, irrespective of the prior distributions (short/long interval), whereas the CMR group selectively added a non-dominant hand response concomitantly to the dominant hand response for one of the priors. The CMR group acquired the two independent priors more quickly, and the divergence between the acquired priors was greater. Facilitation of multiple-prior acquisition was also observed with concomitant vocalization, indicating that this effect is not limited to bimanual interactions. These results demonstrate behavioural contexts that facilitate multiple-prior acquisition while using an identical type of motor response and effector, which can be effective in utilizing Bayesian estimation in daily life.

## 1. Introduction

The brain can improve sensorimotor performance (e.g. hit rates of ball games in daily tasks) by acquiring the prior distribution of a target (e.g. ball speed) and integrating it with sensory signals by Bayes’ rule [1,2]. Behaviour consistent with Bayesian integration has been demonstrated in previous human timing studies [3–5], where the temporal properties of stimuli were sampled from a fixed prior distribution in a given testing session. However, we regularly encounter multiple events in daily tasks (e.g. fastball/slowball), each of which can have unique statistics. Therefore, the brain needs to be able to concurrently acquire multiple prior distributions to efficiently utilize Bayesian estimation in real environments.

Roach *et al*. [6] demonstrated two types of acquisition for two different prior distributions (short and long durations) in a timing task. When participants responded using only a keypress regardless of the priors, they initially acquired a single prior by generalizing over the two distributions (generalization). They eventually acquired the two independent priors after approximately a thousand trials of learning. However, when participants used two distinct types of motor responses (keypress/vocalization) according to the priors, they did not acquire a generalized prior. Instead, they rapidly acquired the two independent priors (motor specificity). A more recent study [7] revealed that participants could acquire the two independent priors with identical motor-response types (keypress) when using two different motor effectors (e.g. hand/foot) according to the priors (body-part specificity). However, in real-world scenarios, different types of motor responses or body parts cannot always be selected for each target state. Bayesian estimation would be more effective in daily tasks if multiple priors could be concurrently acquired, even when using the same motor response type and effector.

In this study, we examined the impact of ‘concomitant motor responses’ (CMRs) on multiple-prior acquisition, motivated by previous psychophysical [8] and neurophysiological [9] studies indicating independent representations between bimanual (i.e. with CMR) and unimanual (i.e. without CMR) movements. A psychophysical study on force field learning in reaching demonstrated limited transfers of force fields between unimanual and bimanual learning within the same arm [8]. This finding suggests that the control processes underlying motor learning differ based on the presence or absence of a concomitant arm movement. In principle, such a distinction could also apply to the acquisition of priors in timing tasks. Roach *et al*. [6] proposed the supplementary motor area (SMA) as a potential neural basis for motor specificity, given that SMA neurons exhibit both time-interval tuning [10] and action selectivity [11]. The SMA also has somatotopy (i.e. body part specificity) [12,13] and contains neurons that respond selectively during either unimanual or bimanual movements [9]. We reasoned that if the SMA supports the acquisition of multiple timing priors, this process would be facilitated by selectively assigning a CMR to one of two prior distributions. This hypothesis was initially tested using the non-dominant hand as the CMR, such that priors were associated with either bimanual (with CMR) or unimanual (without CMR) motor responses (experiment 1). To determine whether the influence of a CMR extends beyond bimanual interactions, we subsequently repeated the study using vocalization as the CMR (experiment 2).

## 2. Methods of experiment 1

### (a) Participants

Thirty-two healthy individuals participated in this study. Participants were randomly assigned to one of the two groups: those with or without a CMR [described in (c)Task]. All participants were naive to the purpose of the experiments and had no prior experience with similar tasks to prevent potential confounding effects from previous exposure.

### (b) Stimuli

Each participant sat on a chair with their head on a chin rest, 87 cm from the cathode-ray tube (CRT) monitor (Sony GDM-F520, Japan; 85 Hz, 21 inch) in a dimly lit, sound-shielded room. We used the NBS Presentation (Neurobehavioral Systems, USA) to generate the stimuli and record participants' responses.

**Figure 1.**
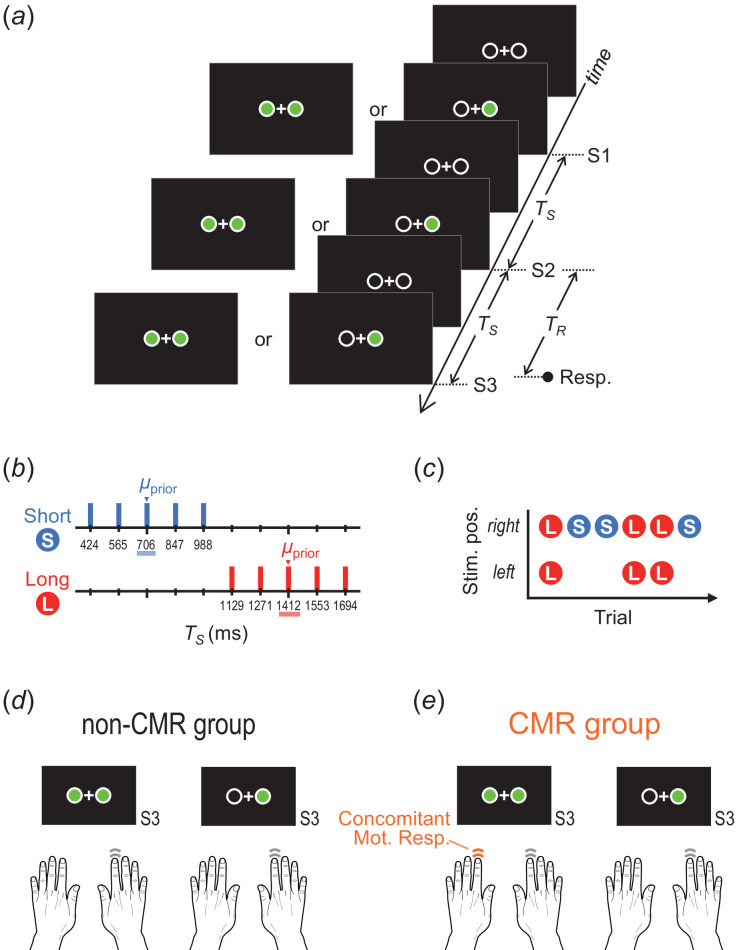
Stimuli and task (experiment 1). (*a*) The stimulus sequence and response in each trial. (*b*) Prior distributions: $T_{S}$ was randomly selected from the short or long prior. (*c*) The short and long priors were assigned to either the both- or one-side stimuli. (*d*) non-CMR group: participants responded using only their dominant hand regardless of the stimuli. (*e*) CMR group: participants added their non-dominant hand response concomitantly with their dominant hand response when stimuli were presented on both sides. $T_{S}$, stimulus time interval; $T_{R}$, response time interval; CMR, concomitant motor response.

In each trial, three sequential stimuli (S1, S2, and S3; each duration: 106 ms) were presented either on one side or both sides of a fixation point (figure 1*a*). The one-sided stimuli were presented on the side of each participant’s dominant hand. The diameter of the frame circles in which the stimuli appeared was 1.1° in the visual angle, and the distance between the centres of the two circles was 2.2°.

The stimulus time intervals ($T_{S}$) between S1 and S2 and between S2 and S3 were identical within each trial. $T_{S}$ was randomly selected from one of the two discrete prior distributions: the short prior (424, 565, 706, 847, and 988 ms; mean [$\mu_{prior}$] = 706 ms) and long prior (1129, 1271, 1412, 1553, and 1694 ms; $\mu_{prior}$ = 1412 ms) (figure 1*b*). In accordance with previous studies [3,4,6,14,15], we used uniform distributions for the priors for two reasons. First, using Gaussian distributions results in lower frequencies for the shorter and longer $T_{S}$ , thereby reducing the statistical reliability of the response data in those $T_{S}$ ranges. Second, even when a uniform distribution is used for the prior, it is assumed that participants acquire a Gaussian distribution [3,6,16], which facilitates the application of the Bayesian estimation model [1,2] to our tasks.

The two priors were assigned to one- or both-side stimuli (figure 1*c*). The prior–stimulus combinations were counterbalanced among the participants: the short (long) prior was assigned to one (both)-side stimuli for half of the participants, and the short (long) prior was assigned to both (one)-side stimuli for the other half. The trial-by-trial order of the priors (i.e. stimulus sides) was randomly selected, with the restriction that $T_{S}$ from the same prior did not repeat for more than four consecutive trials.

### (c) Task

Participants were instructed to press the key(s) using their index finger(s) to coincide with the onset of S3 (coincidence timing task). All participants used their dominant hand for the timing responses in all trials. In addition, they were divided into two groups, with or without a CMR by the non-dominant hand. In the non-CMR group (age: 19.5 ± 1.5 [mean ± standard deviation (s.d.)] [18–22 (min–max)] years; female/male: 6/10; left-/right-hander: 4/12), participants pressed the key only using their dominant hand, regardless of the stimuli sides (figure 1*d*). In the CMR group (age: 19.9 ± 1.7 [18–24] years; female/male: 9/7; left-/right-hander: 4/12), participants also pressed another key using their non-dominant hand concomitantly with the dominant-hand key press when the stimuli appeared on both sides (figure 1*e*). The distance between the centre of the left and right keys was 11.5 cm.

### (d) Procedure

Each participant completed 640 trials (40 trials per session × 16 sessions) of the coincidence timing task. The interval from the onset of S3 to that of S1 in the subsequent trial was 3.1 s. A short beep (0.2 s) was presented 1 s before S1 to alert participants to the start of the trial. Participants took a 1 min break after each session and a 5 min break after every four sessions. When the participants reported fatigue or drowsiness, the break duration was extended.

### (e) Response measurement

We measured the time interval between the onset of S2 and the motor response as the response time interval ($T_{R}$) for each trial (figure 1*a*). We excluded trials with any of the following responses from the analyses: no key press, pressing a key(s) inconsistent with the stimuli or pressing the key(s) twice or more times. In addition, we excluded $T_{R}$ values that were greater or smaller than the mean ± 3 × s.d. for each $T_{S}$ in each trial bin (320 trials per bin) for each participant. We used the $T_{R}$ values of the dominant hand for the analyses. There was no difference in the onsets (or $T_{R}$ values) between the dominant and concomitant non-dominant hand responses (non-dom. − dom.: 0.34 ± 1.45 ms [mean ± standard error of the mean (s.e.m.) across participants], *p* = 0.82, *t*_15_ = 0.24, Pearson’s *r* = 0.061, two-tailed paired *t*-test).

### (f) Theoretical predictions

Applying the Bayesian estimation model [1,2] to the coincidence timing task, ${\overset{-}{T}}_{R}$ (mean among trials of $T_{R}$) is expressed as equation (2.1) [7].


(2.1) $${\overset{-}{T}}_{R}=\frac{\sigma_{\text{prior}}^{2}}{\sigma_{\text{prior}}^{2}+w^{2}T_{S}^{2}}T_{S}+\frac{w^{2}T_{S}^{2}}{\sigma_{\text{prior}}^{2}+w^{2}T_{S}^{2}}\mu_{\text{prior}}$$

where $\mu_{prior}$ and $\sigma_{prior}$ denote the mean and s.d. of the prior distribution, and *w* denotes the Weber fraction. According to scalar variability [17,18], the uncertainty of the sensed $T_{S}$ ($\sigma_{sensed}$) is proportional to $T_{S}$, as expressed by equation (2.2).

(2.2) $$\sigma_{\text{sensed}}=wT_{S}$$

This means that $\sigma_{sensed}$ increases with longer $T_{S}$. Equation (2.1) incorporates the effect of scalar variability.

**Figure 2.**
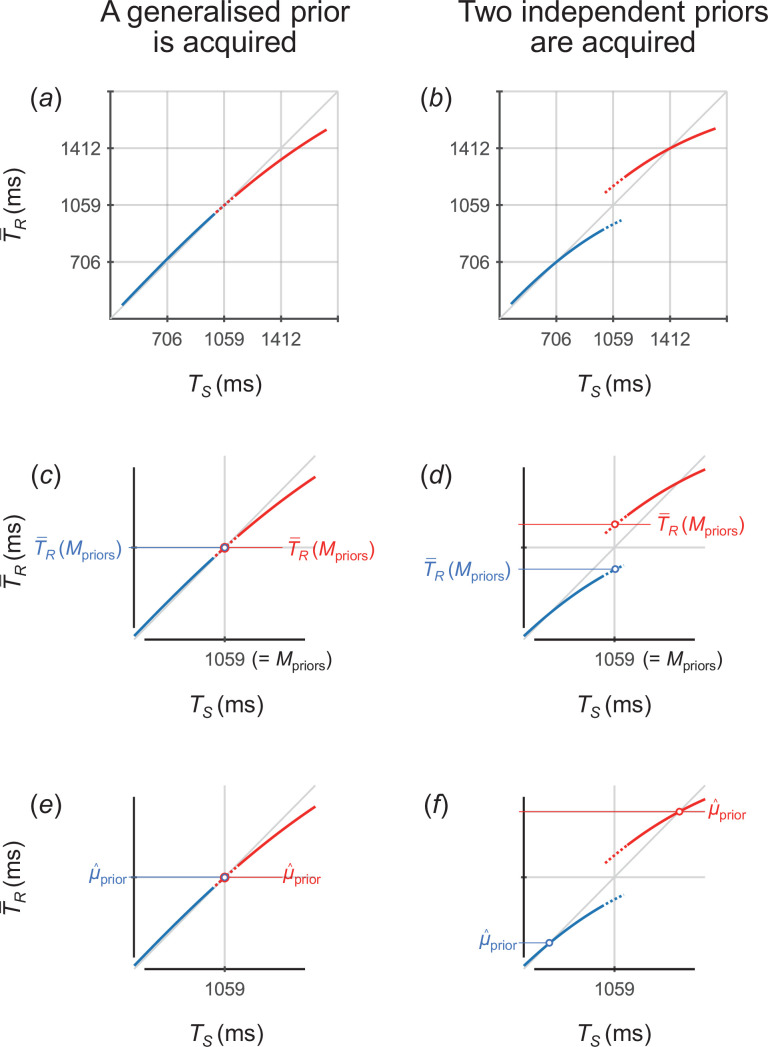
Theoretical predictions and verifications based on the Bayesian estimation model including the effect of scalar variability. (*a*,*b*) Predictions for ${\overset{-}{T}}_{R}$ as a function of $T_{S}$ when participants acquire a single generalized prior (*a*) and when participants acquire the two independent priors (*b*). (*c*,*d*) ${\overset{-}{T}}_{R}\left(M_{priors}\right)$ for the short and long priors when participants acquire a single generalized prior (*c*) and when participants acquire the two independent priors (*d*). (*e*,*f*) ${\hat{\mu }}_{prior}$ for the short and long priors when participants acquire a single generalized prior (*e*) and when participants acquire the two independent priors (*f*). ${\overset{-}{T}}_{R}$, mean across trials of response time intervals; $M_{priors}$, mean over the two prior distributions; ${\hat{\mu }}_{prior}$, mean of the acquired prior distribution.

According to equation (2.1), the ${\overset{-}{T}}_{R}$ × $T_{S}$ function forms a curve with gentler gradients in the longer $T_{S}$ (figure 2*a*,*b*), reflecting that Bayesian timing estimation relies more on the mean of the prior distribution to compensate for greater sensory uncertainty in longer $T_{S}$.

Equation (2.1) predicts the experimental results as follows. If participants acquired a generalized single prior, the curves for the short and long priors should overlap (figure 2*a*), reflecting that their timing estimates are biased towards a common mean over the two prior distributions. Conversely, if participants concurrently acquired short and long priors independently, two distinct curves should appear (figure 2*b*), as their timing estimates are biased towards the respective means of the two prior distributions.

### (g) Verification of theoretical predictions and statistical analyses

We used the $T_{R}$ values for each $T_{S}$ every 320 trials (160 trials per prior) to compute the ${\overset{-}{T}}_{R}$ values for each prior. Next, we fitted the curves based on equation (2.1) to the ${\overset{-}{T}}_{R}$ values as a function of $T_{S}$, using the least-squares method (for details, see electronic supplementary material, methods and table S1).

We calculated ${\overset{-}{T}}_{R}$ at $T_{S}$ = $M_{priors}$ on the fitted curve [${\overset{-}{T}}_{R}\left(M_{priors}\right)$; figure 2*c*,*d*] to verify whether participants acquired two independent priors. $M_{priors}$ denotes the mean over the two prior distributions, which was 1059 ms in experiment 1. If participants acquired a single generalized prior, the ${\overset{-}{T}}_{R}\left(M_{priors}\right)$ values should not differ between the two priors (figure 2*c*). Conversely, if participants concurrently acquired the two independent priors, the ${\overset{-}{T}}_{R}\left(M_{priors}\right)$ values should be greater for the long prior than for the short prior (figure 2*d*).

The normality of the residuals for the difference in ${\overset{-}{T}}_{R}\left(M_{priors}\right)$ between the priors [long − short, $\Delta {\overset{-}{T}}_{R}\left(M_{priors}\right)$] was not rejected (*p* = 0.54, *W* = 0.98, Shapiro–Wilk normality test). Therefore, we tested whether the ${\overset{-}{T}}_{R}\left(M_{priors}\right)$ value was greater for the long prior than for the short prior (i.e. $\Delta {\overset{-}{T}}_{R}\left(M_{priors}\right)$ > 0) per trial bin in each group, using one-tailed paired *t*-tests with Bonferroni correction (significance threshold *α* = 0.05/4 [2 groups × 2 trial bins]).

For the group comparison of the divergences between the two acquired priors, we used the $\Delta {\overset{-}{T}}_{R}\left(M_{priors}\right)$ values. This subtraction method is effective for isolating the effect of interest by reducing interactions among factors [19]. We tested whether the $\Delta {\overset{-}{T}}_{R}\left(M_{priors}\right)$ value was greater for the CMR group than for the non-CMR group using one-tailed Welch’s *t*-tests with Bonferroni correction (*α* = 0.05/2 [2 trial bins]).

In theory, the mean of the acquired prior (${\hat{\mu }}_{prior}$) can be inferred from the point where the ${\overset{-}{T}}_{R}$ × $T_{S}$ curve intersects the unity line (i.e. points where ${\overset{-}{T}}_{R}$ = $T_{S}$) (figure 2*e*,*f*). However, in practice, the estimation of this intersection is sensitive to idiosyncratic responses (e.g. overshoots, undershoots and slopes steeper than unity). In 17.2% of the curve fittings, the ${\hat{\mu }}_{prior}$ values were less than the minimum (424 ms) or greater than the maximum (1693 ms) of $T_{S}$, and some values were highly implausible (e.g. < −10 000 ms, >10 000 ms).

To address this issue, we calculated the ${\hat{\mu }}_{prior}$ values using the grand-averaged ${\overset{-}{T}}_{R}$ values (i.e. means across participants), where the individuals’ idiosyncratic responses were averaged out. In both theory and practice, ${\hat{\mu }}_{prior}$ can be accounted for by ${\overset{-}{T}}_{R}\left(M_{priors}\right)$ [7]. Therefore, these two values can compensate for one another.

Moreover, we applied the permutation method to the group comparisons of $\begin{array}{l} \Delta {\hat{\mu }}_{prior} \end{array}$ (difference in ${\hat{\mu }}_{prior}$ between the priors [long − short]). We first randomly permuted the individuals’ ${\overset{-}{T}}_{R}$ sequences against $T_{S}$ for each trial bin across the non-CMR and CMR groups. Subsequently, we averaged the permuted ${\overset{-}{T}}_{R}$ sequences across 16 individuals for each trial bin in each group. We generated 10 000 sets of grand-averaged permutation ${\overset{-}{T}}_{R}$ sequences for each trial bin in each group (40 000 sets in total) and conducted curve fittings on them to obtain the ${\hat{\mu }}_{prior}$ values. Notably, no irregular ${\hat{\mu }}_{prior}$ value emerged from the permutation process. Finally, we calculated the differences in the permutation $\begin{array}{l} \Delta {\hat{\mu }}_{prior} \end{array}$ values between the groups (CMR − non-CMR), and counted those that were greater than the original ones (*n*_perm > org_) to compute the permutation *p* values (*n*_perm > org_/10 000) for each trial bin (*α* = 0.05/2, Bonferroni correction).

We used Pearson’s *r* as the effect size index for the *t*-tests, as *r* can also be used for Wilcoxon signed-rank tests that were used in supplementary results (electronic supplementary material).

In addition, we calculated Akaike weights [20] based on the Akaike information criterion [21] for two types of fittings based on the acquisition of two independent priors (two-prior model) and that of one generalized prior (one-prior model; for details, see electronic supplementary material, methods and table S2). Akaike weights provide an index of the relative strength of evidence for the models under consideration, such that higher weights for the two-prior model signify stronger evidence for the acquisition of two independent priors.

## 3. Results of experiment 1

**Figure 3.**
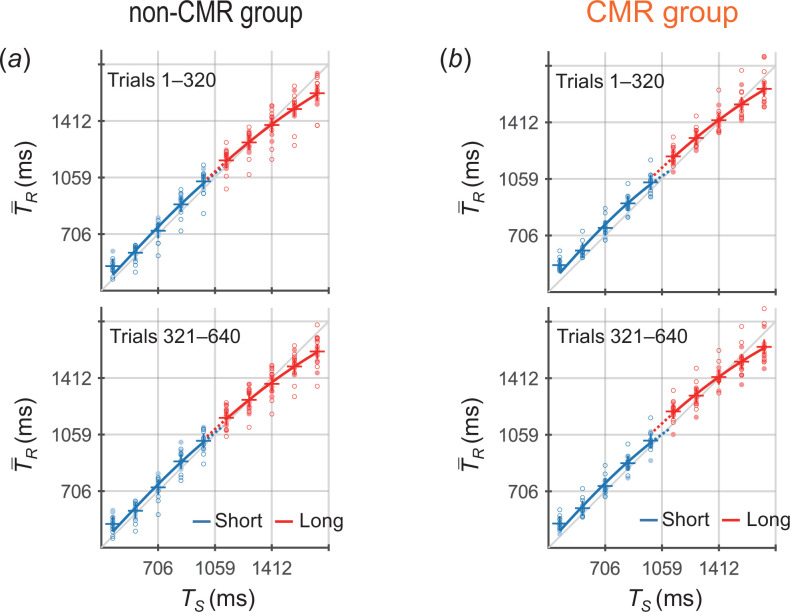
${\overset{-}{T}}_{R}$ values across participants as a function of $T_{S}$ in trials 1–320 (upper) and 321–640 (lower) for (*a*) the non-CMR group and (*b*) the CMR group (experiment 1). Blue and red markers represent the values for the short and long priors, respectively. The circle markers represent the ${\overset{-}{T}}_{R}$ values of each participant, with open and closed circles indicating cases where the short (long) prior distribution was assigned to the one (both)-side stimuli and to the both (one)-side stimuli, respectively. The plus markers represent the grand-averaged ${\overset{-}{T}}_{R}$ values (means across participants). Blue and red lines represent the curves fitted to the grand-averaged ${\overset{-}{T}}_{R}$ values using equation (2.1) for the short and long priors, respectively.

Figure 3 shows the ${\overset{-}{T}}_{R}$ values across participants as a function of $T_{S}$. In the non-CMR group (figure 3*a*), the ${\overset{-}{T}}_{R}$ × $T_{S}$ curves for the short and long priors approximately overlapped in trials 1–320, consistent with the theoretical prediction that a single generalized prior was acquired (figure 2*a*) and then a slight gap appeared between the two priors in trials 321–640. In contrast, in the CMR group (figure 3*b*), the ${\overset{-}{T}}_{R}$ × $T_{S}$ curves for the two priors diverged in both trials 1–320 and 321–640, which is consistent with the prediction that two independent priors were acquired (figure 2*b*).

**Figure 4.**
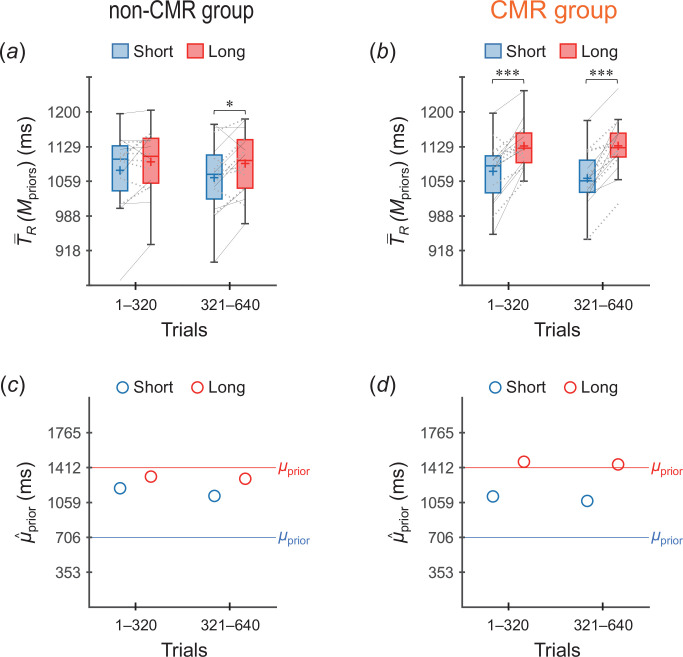
Verifications of whether two independent priors were acquired (experiment 1). (*a*,*b*) ${\overset{-}{T}}_{R}\left(M_{priors}\right)$ values across participants (box plots) calculated per 320 trials (160 trials/prior) for (*a*) the non-CMR group and (*b*) the CMR group. Blue and red boxes represent the values for the short and long priors, respectively. The plus sign in each box indicates the mean across participants. The left and right ends of each line indicate individual values for the short and long priors, respectively, with thin-black and dotted-grey lines indicating cases where the short (long) prior distribution was assigned to the one (both)-side stimuli and to the both (one)-side stimuli, respectively. *p < 0.05/4, ***p < 0.005/4 [22]. (*c*,*d*) ${\hat{\mu }}_{prior}$ values inferred using the grand-averaged ${\overset{-}{T}}_{R}$ values per 320 trials for (*c*) the non-CMR group and (*d*) the CMR group. The blue and red open circles represent the values for the short and long priors, respectively.

Figure 4*a*,*b* shows ${\overset{-}{T}}_{R}\left(M_{priors}\right)$ values across participants. In the non-CMR group (figure 4*a*), the ${\overset{-}{T}}_{R}\left(M_{priors}\right)$ values were not significantly different between the two priors in trials 1–320 (*p* = 0.034 > 0.05/4, *t*_15_ = 1.97, *r* = 0.45), although the value was significantly greater for the long prior than for short prior in trials 321–640 (*p* = 0.0073 < 0.05/4, *t*_15_ = 2.76, *r* = 0.58). Meanwhile, in the CMR group (figure 4*b*), the ${\overset{-}{T}}_{R}\left(M_{priors}\right)$ values were significantly greater for the long prior than for the short prior in both trials 1–320 (*p* = 1.0 × 10^−4^ < 0.05/4, *t*_15_ = 4.88, *r* = 0.78) and trials 321–640 (*p* = 4.3 × 10^−7^ < 0.05/4, *t*_15_ = 8.00, *r* = 0.90), which are consistent with the case of acquiring two independent priors (figure 2*d*).

Figure 4*c,d* shows ${\hat{\mu }}_{prior}$ values calculated using the grand-averaged ${\overset{-}{T}}_{R}$ values. The ${\hat{\mu }}_{prior}$ values were generally greater for the long prior than for the short prior. However, the divergence in ${\hat{\mu }}_{prior}$ between the two priors was greater for the CMR group (figure 4*d*) than for the non-CMR group (figure 4*c*), which is consistent with the means across participants of the ${\overset{-}{T}}_{R}\left(M_{priors}\right)$ values (plus markers in figure 4*a,b*).

**Figure 5.**
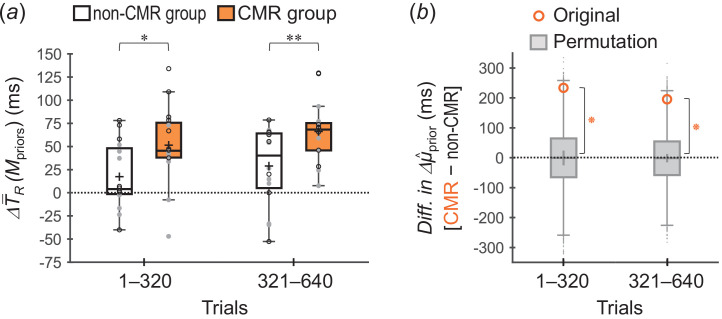
Group comparisons for divergences between the two acquired priors (experiment 1). (*a*) $\Delta {\overset{-}{T}}_{R}\left(M_{priors}\right)$ values across participants (box plots) for the non-CMR group (white-faced boxes) and CMR group (orange-faced boxes). $\Delta {\overset{-}{T}}_{R}\left(M_{priors}\right)$ was calculated by subtracting ${\overset{-}{T}}_{R}\left(M_{priors}\right)$ for the short prior from that for the long prior. The plus marker in each box indicates the mean across participants. Each small circle represents the individual value, with opened-black and closed-grey circles indicating that the short (long) prior distribution was assigned to the one (both)-side stimuli and to the both (one)-side stimuli, respectively. *p < 0.05/2, **p < 0.01/2. (*b*) Differences in $\begin{array}{l} \Delta {\hat{\mu }}_{prior} \end{array}$ between the groups (CMR − non-CMR) for the original data (opened-orange circles) and permutation data (grey-faced boxes). $\begin{array}{l} \Delta {\hat{\mu }}_{prior} \end{array}$ was calculated by subtracting ${\hat{\mu }}_{prior}$ for the short prior from that for the long prior. The plus sign in each box indicates the mean of the permutation data. ⁕Permutation *p* < 0.05/2.

Figure 5*a* shows $\Delta {\overset{-}{T}}_{R}\left(M_{priors}\right)$ values across participants for the non-CMR and CMR groups. The $\Delta {\overset{-}{T}}_{R}\left(M_{priors}\right)$ values were greater for the CMR group than for the non-CMR group in both trials 1–320 (*p* = 0.0096 < 0.05/2, *t*_29.1_ = 2.48, *r* = 0.42) and trials 321–640 (*p* = 0.0046 < 0.05/2, *t*_28.5_ = 2.79, *r* = 0.46). The results indicated that the divergence between the acquired priors was greater in the CMR group than in the non-CMR group.

Figure 5*b* shows the group comparisons of $\Delta {\hat{\mu }}_{prior}$ using the permutation method. The differences in $\Delta {\hat{\mu }}_{prior}$ between the groups (CMR − non-CMR) were significantly greater for the original data than for the permutation data in both trials 1–320 (permutation *p* = 0.0055 < 0.05/2) and trials 321–640 (permutation *p* = 0.0079 < 0.05/2). The results further supported that the divergence between the acquired priors was greater in the CMR group than in the non-CMR group.

Moreover, Akaike weights supported the acquisition of two independent priors in the CMR group. For the non-CMR group, the Akaike weights were smaller for the two-prior model than for the one-prior model in both trial bins (two/one: 0.12/0.88 for trials 1–320, 0.15/0.85 for trials 321–640). However, for the CMR group, the Akaike weights were greater for the two-prior model than for the one-prior model in both trial bins (two/one: 0.69/0.31 for trials 1–320, 0.83/0.17 for trials 321–640).

## 4. Methods of experiment 2

**Figure 6.**
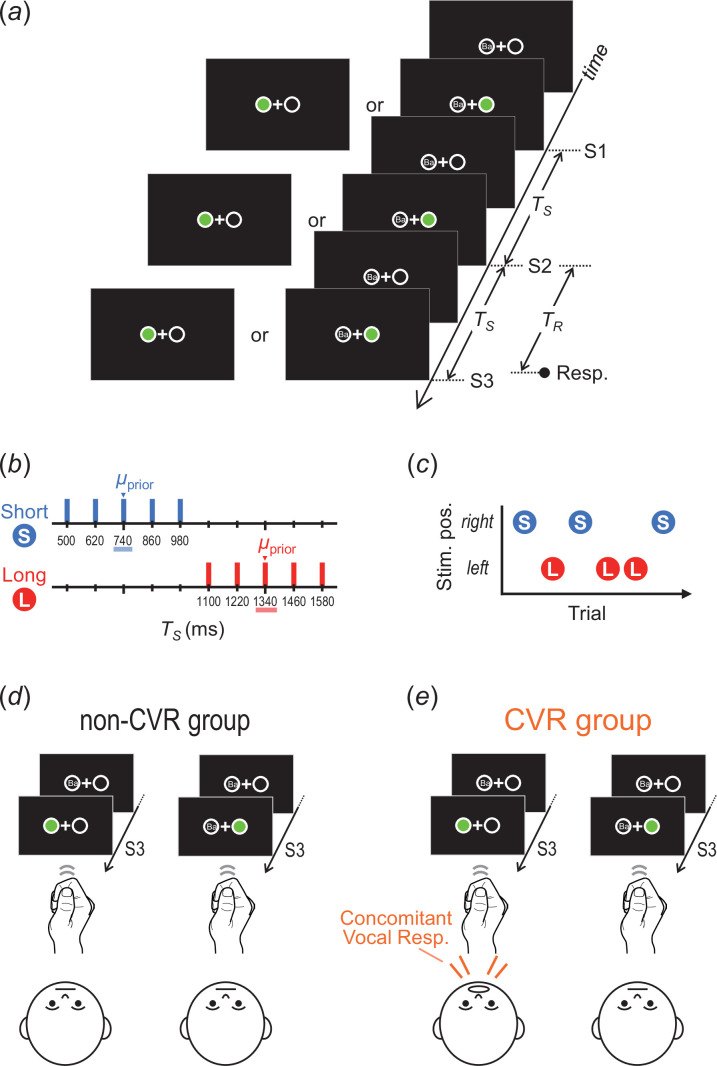
Stimuli and task (experiment 2). (*a*) The stimulus sequence and response in each trial. (*b*) Prior distributions. (*c*) The short and long priors were assigned to either *right* or *left* stimuli. (*d*) non-CVR group: participants responded using only their dominant hand regardless of the stimuli. (*e*) CVR group: participants vocalized ‘Ba’ concomitantly with their dominant hand response when stimuli were presented on the side labelled ‘Ba’. CVR, concomitant vocal response.

In experiment 1, the selective assignment of the CMR by the non-dominant hand facilitated the acquisition of the two independent priors. This finding raises the question of whether the facilitatory effect of CMR is specific to bimanual interactions or generalizes to other types of motor responses. To answer this, we repeated the experiment using vocalization as a CMR (concomitant vocal response, CVR; figure 6), which is often observed in daily behaviours such as sports. General methods were similar to those of experiment 1, except for the following aspects.

### (a) Stimuli

Stimuli were presented on a CRT display (Eizo T566, Japan, 100 Hz, 17 inch), which was positioned 51 cm from the participants’ heads to equalize the visual-angle sizes of stimuli with those in experiment 1. S1, S2, and S3 were presented on either the right or left side of the fixation point (figure 6*a*). In the background, ‘Ba’ was labelled in either the right- or left-side frame circles where the stimuli appeared. The side displaying ‘Ba’ was counterbalanced between the participants. $T_{S}$ was randomly selected from the short prior (500, 620, 740, 860 and 980 ms; $\mu_{prior}$ = 740 ms) or long prior (1100, 1220, 1340, 1460 and 1580 ms; $\mu_{prior}$ = 1340 ms) (figure 6*b*), where $M_{priors}$ = 1040 ms. These two priors were assigned to the right- or left-side stimuli (figure 6*c*).

### (b) Task

Participants (*n* = 32, naive and inexperienced) were instructed to press a hand-held button with their dominant hand (thumb) to coincide with the onset of S3. During the task, the participants placed their dominant hand on a table along the median plane. They were classified into two groups, with or without a CVR. In the non-CVR group (age: 20.1 ± 1.1 [18–22] years; female/male: 3/13; left-/right-hander: 0/16), participants only pressed the button with their dominant hand, regardless of the stimuli sides (figure 6*d*). In the CVR group (age: 20.7 ± 2.1 [18–25] years; female/male: 2/14; left-/right-hander: 3/13), participants vocalized ‘Ba’ concomitantly with the dominant-hand response when the stimuli appeared on the side labelled ‘Ba’ (figure 1*e*). Therefore, the CVR was selectively assigned to one of the two priors. Vocal and hand responses were recorded using a USB response pad with a voice key and hand-held response button (Black Box Toolkit, UK). The onsets of the concomitant vocal responses were 91.0 ± 10.0 ms later than those of the dominant hand responses (*p* = 1.8 × 10^−7^, *t*_15_ = 9.06, *r* = 0.92, two-tailed paired *t*-test).

### (c) Statistical analyses

We conducted the same analyses as those performed in experiment 1. As in experiment 1, the normality of residuals was not rejected for $\Delta {\overset{-}{T}}_{R}\left(M_{priors}\right)$ between the priors (*p* = 0.26, *W* = 0.98, Shapiro–Wilk normality test). In 25.0% of the curve fittings, the ${\hat{\mu }}_{prior}$ values were less than the minimum (500 ms) or greater than the maximum (1580 ms) of $T_{S}$, For the permutation analyses, irregular ${\hat{\mu }}_{prior}$ values were obtained for the short prior in 0.435% of the permutation data. The subsequent analyses were performed after removing the irregular permutation ${\hat{\mu }}_{prior}$ values. However, calculating the permutation *p* values with the irregular ${\hat{\mu }}_{prior}$ values included did not affect the statistical conclusions (see also the legend of figure 9).

## 5. Results of experiment 2

**Figure 7.**
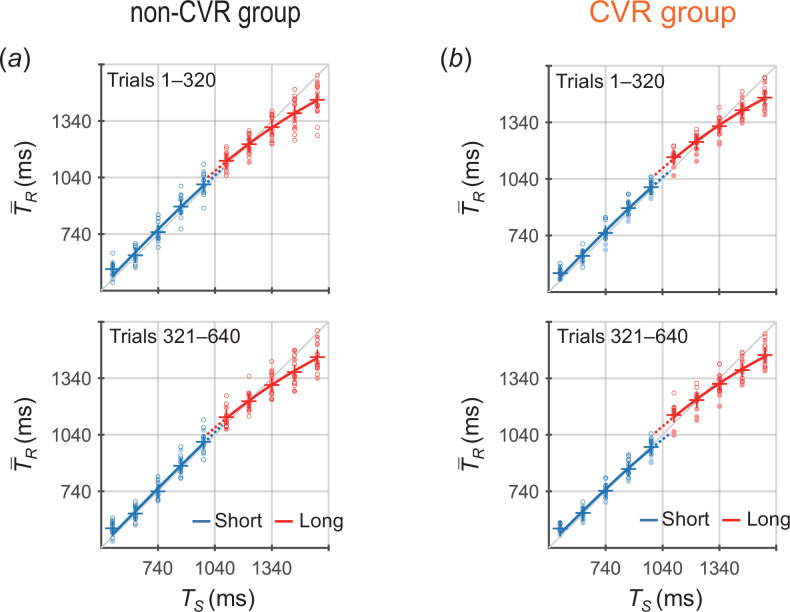
${\overset{-}{T}}_{R}$ values across participants as a function of $T_{S}$ for (*a*) the non-CVR group and (*b*) the CVR group (experiment 2). The representations of the marker and lines are similar to those in figure 3. The opened and closed circle markers indicate the individual ${\overset{-}{T}}_{R}$ values in cases where the short (long) prior distribution was assigned to the side labelled no ‘Ba’ (labelled ‘Ba’) and to that labelled ‘Ba’ (labelled no ‘Ba’), respectively.

The ${\overset{-}{T}}_{R}$ × $T_{S}$ curves exhibited slight gaps between the two priors in trials 1–320 and 321–640 for the non-CVR group (figure 7*a*). Meanwhile, relatively large divergences were found between the two priors for the CVR group (figure 7*b*).

**Figure 8.**
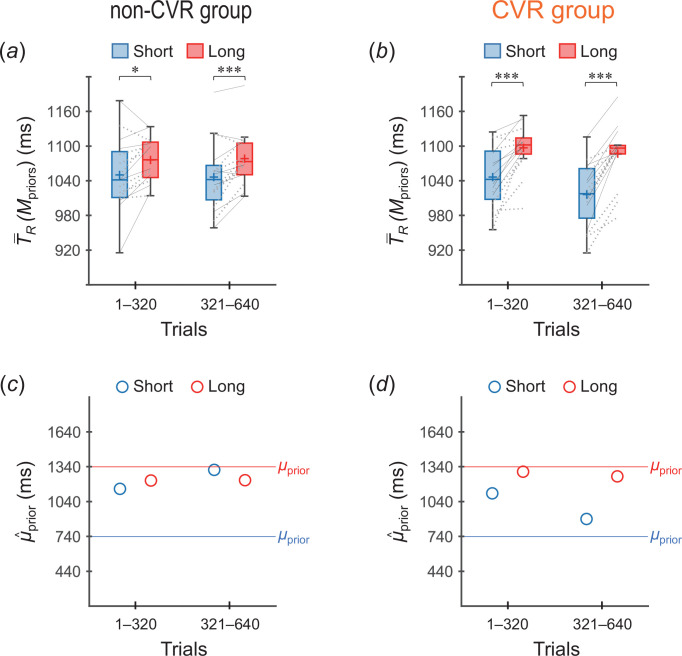
Verifications of whether two independent priors were acquired (experiment 2). (*a*,*b*) ${\overset{-}{T}}_{R}\left(M_{priors}\right)$ values across participants for (*a*) the non-CVR group and (*b*) the CVR group. *p < 0.05/4, ***p < 0.005/4. (*c*,*d*) ${\hat{\mu }}_{prior}$ values inferred using the grand-averaged ${\overset{-}{T}}_{R}$ values for (*c*) the non-CVR group and (*d*) the CVR group. The representations of the markers, lines and boxes are similar to those in figure 4. The thin-black and dotted-grey lines in (*a*) and (*b*) indicate the individual ${\overset{-}{T}}_{R}\left(M_{priors}\right)$ values in cases where the short (long) prior distribution was assigned to the side labelled no ‘Ba’ (labelled ‘Ba’) and to that labelled ‘Ba’ (labelled no ‘Ba’), respectively.

The ${\overset{-}{T}}_{R}\left(M_{priors}\right)$ values were significantly greater for the long prior than for the short prior in both trial bins in the non-CVR group (trials 1–320: *p* = 0.0052 < 0.05/4, *t*_15_ = 2.92, *r* = 0.60; trials 321–640: *p* = 3.0 × 10^−4^ < 0.05/4, *t*_15_ = 4.32, *r* = 0.74) (figure 8*a*) and in the CVR group (trials 1–320: *p* = 6.3 × 10^−5^ < 0.05/4, *t*_15_ = 5.12, *r* = 0.80; trials 321–640: *p* = 9.4 × 10^−8^ < 0.05/4, *t*_15_ = 9.03, *r* = 0.92) (figure 8*b*).

Figure 8*c*,*d*, shows that the divergence in ${\hat{\mu }}_{prior}$ between the two priors was greater for the CVR group than for the non-CVR group. Therefore, the grand-averaged ${\hat{\mu }}_{prior}$ values were generally consistent with the means across participants for ${\overset{-}{T}}_{R}\left(M_{priors}\right)$ values (plus markers in figure 8*a*,*b*) although ${\hat{\mu }}_{prior}$ was slightly greater for the short prior than for the long prior in trials 321–640 for the non-CVR group.

**Figure 9.**
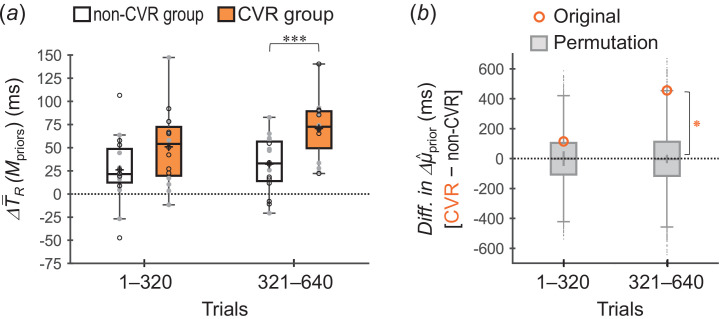
Group comparisons for divergences between the two acquired priors (experiment 2). (*a*) $\Delta {\overset{-}{T}}_{R}\left(M_{priors}\right)$ values across participants for the non-CVR group and CVR group. ***p < 0.005/2. (*b*) Differences in $\begin{array}{l} \Delta {\hat{\mu }}_{prior} \end{array}$ between the groups (CVR − non-CVR) for the original data and permutation data. ⁕Permutation *p* < 0.05/2. The box plots for the permutation data were created after removing the irregular permutation ${\hat{\mu }}_{prior}$ values. Notably, including the irregular permutation ${\hat{\mu }}_{prior}$ values does not affect the statistical conclusions (trials 1–320: permutation *p* = 0.23, trials 1–320: permutation *p* = 0.0086 < 0.05/2). The representations of the markers, lines and boxes are similar to those in figure 5. The opened-black and closed-grey circles in (*a*) indicate the individual $\Delta {\overset{-}{T}}_{R}\left(M_{priors}\right)$ values in cases where the short (long) prior distribution was assigned to the side labelled no ‘Ba’ (labelled ‘Ba’) and to that labelled ‘Ba’ (labelled no ‘Ba’), respectively.

As shown in figure 9*a*, the $\Delta {\overset{-}{T}}_{R}\left(M_{priors}\right)$ values across participants were significantly greater for the CVR group than for the non-CVR group in trials 321–640 (*p* = 6.1 × 10^−4^ < 0.05/2, *t*_29.9_ = 3.57, *r* = 0.55), although the difference was not significant in trials 1–320 (*p* = 0.037 > 0.05/2, *t*_29.7_ = 1.85, *r* = 0.32).

Similar results were obtained in the group comparisons of $\begin{array}{l} \Delta {\hat{\mu }}_{prior} \end{array}$ using the permutation method (figure 9*b*). The difference in $\begin{array}{l} \Delta {\hat{\mu }}_{prior} \end{array}$ between the groups (CVR − non-CVR) was significantly greater for the original data than for the permutation data in trials 321–640 (permutation *p* = 0.0056 < 0.05/2), although the difference was non-significant in trials 1–320 (permutation *p* = 0.23).

These group comparisons indicated that the divergence between the acquired priors was greater in the CVR group than in the non-CVR group during the latter half of the trials in experiment 2.

In addition, Akaike weights supported the acquisition of two independent priors in the CVR group. For the non-CVR group, the Akaike weights were smaller for the two-prior model than for the one prior model in trials 1–320 (two/one: 0.42/0.58), although they were greater for the two-prior model in trials 321–640 (two/one: 0.89/0.11). In contrast, for the CMR group, the Akaike weights were completely greater for the two-prior model than for the one-prior model in both trial bins (two/one: 0.999/0.001 for trials 1–320, 0.9999/0.0001 for trials 321–640).

## 6. Discussion

### (a) Selective assignment of concomitant motor responses facilitates multiple-prior acquisition

The current results support the hypothesis that the selective assignment of concomitant motor responses facilitates the concurrent acquisition of multiple priors. When participants selectively added a non-dominant hand response concomitantly with the dominant hand response according to the priors, they acquired the two independent priors more quickly and the divergence between them was greater than when the concomitant motor response was not available (experiment 1). Moreover, this facilitation was not limited to bimanual interactions. When participants selectively vocalized concomitantly with the dominant hand response according to the priors, the two independent priors were acquired; in the latter half of the trials, the divergence between the acquired priors was greater than when the concomitant vocal response was not available (experiment 2).

Previous studies have shown that the use of different types of motor responses [6] or effectors [7] can promote concurrent acquisition of multiple priors. Our findings extend these works by showing that more subtle manipulations of behavioural context also facilitate multiple-prior acquisition, even when a single type of motor response and effector is being used. While laboratory studies often rely on simple behavioural responses, natural behaviour is more complex, often involving multiple motor actions by different effectors [23–25]. As this complexity increases the set of concomitant motor responses, it, therefore, provides a potential solution to the problem of distinguishing and acquiring multiple different priors in real-world environments.

### (b) A possible neural basis for multiple-prior acquisition

A motivating factor for our initial hypothesis was a neurophysiological report [9] that found selective neuronal activity between unimanual movement (i.e. without concomitant movement) and bimanual movement (i.e. with concomitant movement) in the SMA, which has been proposed as a possible neural basis for multiple-prior acquisition [6,7]. Based on this, we designed experiment 1 and the results supported our hypothesis.

Moreover, the results of experiment 2 are potentially consistent with the involvement of the SMA in multiple-prior acquisition. A functional magnetic resonance imaging study [26] reported that the SMA was activated when healthy participants performed a hand task and when they performed a combined hand–speech task. Notably, the SMA activity during the combined hand–speech task was found at a relatively superior region compared with that during the hand task, which may reflect selective activity between hand movements with or without vocalization.

Thus, both previous and current findings are consistent with the proposal that the SMA is the neural basis for multiple-prior acquisition [6,7]. However, this proposal remains speculative at this stage. Neuroimaging or neurophysiological data are needed to confirm this hypothesis in future studies.

### (c) Differences between using the non-dominant hand and vocalization for concomitant motor responses

Some differences between the results for the CMR (experiment 1) and CVR (experiment 2) groups are worth noting. The effects of concomitant motor responses were evident from trials 1–320 for the CMR group (figure 5), whereas they emerged later in trials 321–640 for the CVR group (figure 9). Additionally, in the CMR group, the ${\hat{\mu }}_{prior}$ values for the short prior were continuously biased toward the long prior (figure 4*d*). In contrast, in the CVR group, although a similar bias appeared, the ${\hat{\mu }}_{prior}$ for the short prior shifted towards the actual $\mu_{prior}$ (740 ms) in trials 321–640 (figure 8*d*). These observations were supported by supplementary statistical tests on the ${\overset{-}{T}}_{R}\left(M_{priors}\right)$ values (see electronic supplementary material, results).

Similar differences were observed between participants who used their right and left hands and those who used their dominant index and middle fingers in a previous study [7]. Thus, two independent priors were acquired relatively quickly, but the acquired short prior remained biased toward the long prior for an extended period when both hands were used. Although the actual underlying mechanism is unclear at this stage, various neural interactions occur during bimanual movements [27,28], which may influence the two-prior acquisition.

### (d) A possible effect of multiple cues for priors

Additional unexpected results were also obtained during our experiments. The ${\overset{-}{T}}_{R}\left(M_{priors}\right)$ values indicated that the two independent priors were also acquired in the control groups, who responded using only their dominant hands (figures 4*a* and 8*a*), though it should be noted that the divergence between the priors was consistently smaller than in the groups with concomitant motor responses and that additional analyses (Akaike weights and ${\hat{\mu }}_{prior}$) did not always support this conclusion. In contrast, a previous study using similar timing tasks [7] did not show any difference in ${\overset{-}{T}}_{R}\left(M_{priors}\right)$ between the short and long priors within 640 trials in the control group.

Differences in findings between the current and previous studies could be explained by the availability of multiple cues for the priors. In the previous study, an identical stimulus sequence was presented on the right or left sides; therefore, the task stimuli provided only a spatial cue to distinguish the priors. In contrast, in experiment 1, one- and two-sided stimuli were presented in relation to the dominant hand side and both sides, respectively (figure 1*a*), providing numerical and spatial cues to distinguish the priors. In experiment 2, ‘Ba’ was labelled on either the left or right side (figure 6*a*), providing literal and spatial cues to distinguish the priors.

These increases in cues for priors may have facilitated multiple-prior acquisition. In previous studies, aftereffects in tactile temporal order judgment (TOJ) were consistent with the Bayesian estimation model [29,30], and concurrent aftereffects of two opposing tactile orders on TOJ were greater when two cues (colours and retinal positions of stimuli) were used to indicate the orders than when one of the cues was used [31]. This result supports the explanation that multiple cues enhance the acquisition of multiple priors.

### (e) A possible effect of the prior–stimulus combinations

In experiment 1 (figure 5*a*), the individuals’ $\Delta {\overset{-}{T}}_{R}\left(M_{priors}\right)$ values tended to be higher when the short (long) prior was assigned to the one (both)-side stimuli (opened-black circles) than when the prior–stimulus combinations were reversed (closed-grey circles). Additional analysis revealed a marginal effect of prior–stimulus combination (*p* = 0.051; see electronic supplementary material, results and table S3). In contrast, in experiment 2 (figure 9*a*), no effect of the prior–stimulus combination was found (*p* = 0.85). The effect of the prior–stimulus combination in experiment 1 was not statistically significant but non-negligible. This could be associated with the compatibility between priors (time) and cues (numerosity). The judgements of temporal and numerous magnitudes interact with each other [32–34]. The time–numerosity compatibility was stronger for the short–one and long–both (two) combinations than for the short–both and long–one combinations. Compatible prior–cue assignments may facilitate the distinction between priors and facilitate multiple-prior acquisition.

## 7. Conclusions and perspectives

The current study demonstrated that participants could more effectively acquire two independent priors in a coincidence timing task by selectively adding concomitant motor responses, even when using an identical type of motor response and effector. These findings advance our psychophysical understanding and neurophysiological insights into multi-prior acquisition and have implications for our daily behaviours such as sports [35–38]. For instance, tennis players who selectively grip with their non-dominant hand or shout according to ball speed may optimize their dominant-hand timing when receiving the ball. Our findings on multiple-prior acquisition contribute to extending the Bayesian approach to understanding and improving daily behaviours.

## Data Availability

Data and codes to reproduce the results are provided in the electronic supplementary material [39].
